# Improved performance of soy protein adhesive with melamine–urea–formaldehyde prepolymer

**DOI:** 10.1039/d1ra00850a

**Published:** 2021-08-09

**Authors:** Ke Jiang, Zhenghui Lei, Maoyu Yi, Wenxin Lv, Mingwei Jing, Qiaoling Feng, Hailu Tan, Yuzhu Chen, Hui Xiao

**Affiliations:** College of Forestry, Sichuan Agricultural University Chengdu 611130 Sichuan China; Key Laboratory of Wood Industry and Furniture Engineering, Sichuan Provincial Department of Education, Sichuan Agricultural University Chengdu 611130 Sichuan China shawwe@126.com +86-028-86291456

## Abstract

In recent years, soy protein adhesive, as an environmentally friendly bio-based adhesive, has attracted extensive attention. In this study, in order to ameliorate the bonding quality of soy protein isolate (SPI) adhesive, the melamine–urea–formaldehyde prepolymer (MUFP) was synthesized, and different amounts of it were introduced into the SPI adhesive as a cross-linking agent. Fourier transform infrared (FT-IR) spectroscopy, gel permeation chromatography (GPC), thermogravimetric analyze (TGA), and scanning electron microscopy (SEM) were used to analysis the mechanism of modification. The results of plywood test indicated that the wet bonding strength of the adhesives was first increased and then decreased with an increase in the amount of MUFP additive. FT-IR, TGA, and SEM tests suggested that the introduction of MUFP could promote the establishment of a cross-linking structure in the cured adhesive layer to improve the bonding quality of adhesives, but presence of excessive MUFP could introduce hydrophilic groups and adversely affect water resistance.

## Abstract 2

In recent years, soy protein adhesive, as an environmentally friendly bio-based adhesive, has attracted extensive attention.

## Graphical abstract

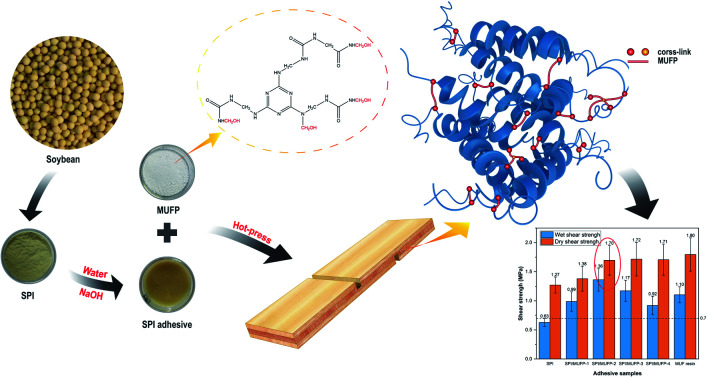

## 1. Introduction

In recent years, with the continuous improvement of the public's environmental awareness, renewable environmentally-friendly bio-based adhesives have garnered great research attention. Soy-based adhesive has attracted more and more attention due to its advantages of easy degradation, renewable, and rich in yield.^1,2^ However, soy-based adhesives have extremely low wet bonding performance due to the fact that the bonding force is only provided by intermolecular hydrogen bonds and the physical entanglement from the peptide chains, limiting the application of soy-based adhesives. In various soy-based adhesives, the soy protein adhesive has the best performance but still inferior to petroleum-based resins.^3,4^ Therefore, many physical and chemical methods have been studied in order to enhance the water resistance and bonding strength of soy-based adhesive.

Protein denaturation methods such as heat treatment,^5,6^ acid–alkali treatment,^7,8^ and surfactant treatment^9,10^ can improve the water resistance of the adhesive *via* expanding the tertiary and quaternary structure of soy protein to expose the hydrophobic groups. A research showed that moderate hot-alkali treatment (pH = 9 and 50 °C) of protein can improve the water resistance of adhesives without corroding the wood.^8^ However, these denaturation methods can only be used as pretreatment due to the low degree of improvement in the bonding strength of the adhesive. Protein modification methods such as cross-linking modification,^11^ nanomaterial treatment,^12,13^ and grafting modification^14,15^ can further improve the bonding performance of the adhesive after pretreatment. In the above modification methods, the cross-linking modification is the most effective improvement method, which promote the formation of a stable three-dimensional network structure of soy protein through chemical cross-linking reaction.^16–18^ Compounds with two or more functional groups which can react with the reactive groups of proteins are usually used as cross-linkers,^19^ such as epoxy compounds,^20^ aldehyde and its derivatives,^21^ waterborne polyurethane,^22^ tannins,^23^ lignin-based resin,^24^*etc.* Luo *et al.* employed 6% neopentyl glycol diglycidyl ether to increase the wet shear strength of the soy-based adhesive to 1.12 MPa.^25^ The Soyad™ adhesive, a soy-based adhesive modified by polyamide epichlorohydrin resin, is widely used as a commercial adhesive.^26^ However, the high-efficiency cross-linkers are usually expensive, which will further increase the production cost of soy protein adhesives and limit the application of adhesives. In order to reduce the cost of cross-linker, Fan *et al.*^27^ used 40% inexpensive MUF resin to improve the wet shear strength of the soy-based adhesive to 1.16 MPa. The results indicated that the increase was attributed to the cross-linking reaction between the hydroxymethyl groups from the MUF resin and the amino groups from the soy protein. Moreover, some studies have successfully copolymerized soy protein hydrolysate with amino formaldehyde resins to prepare biodegradable resins, which further confirmed the reaction between hydroxymethyl and soy protein.^28,29^ However, the modification efficiency of these amino formaldehyde resins is low due to their large molecular weight and poor reactive groups. High amount (usually greater than 10% of the total mass of the adhesive) of synthetic resin additive can effectively improve the performance of the soy-based adhesive, but it also causes a waste of non-renewable petroleum resources.^30,31^

In this study, in order to solve the contradiction between the cost of modification and the consumption of non-renewable resources, an efficient and inexpensive cross-linker melamine–urea–formaldehyde prepolymer (MUFP) was developed. Although the raw materials of MUFP and MUF resin are the same, the hydroxymethylated prepolymer has a lower molecular weight and more reactive groups than amino formaldehyde resin, which ensures that it can react with soy protein to a greater degree.^32,33^ The water resistance and bonding strength of soy protein adhesive can be improved with addition of less amount of hydroxymethylated prepolymer.^34^ Furthermore, the performance of soy protein adhesive modified with different amounts of MUFP additive were studied. Fourier transform infrared (FT-IR) spectroscopy, gel permeation chromatography (GPC), thermogravimetric analyze (TGA), and scanning electron microscopy (SEM) were used to analysis the mechanism of improvement of water resistance and bonding strength of modified adhesives.

## 2. Materials and methods

### 2.1. Materials

SPI (91% protein, 2.5% fat, and 0.8% carbohydrate) was acquired from Linyi Shansong Biological Products Co., Ltd. (Shandong, China). Melamine (analytically pure), urea (analytically pure), formaldehyde solution (37 wt%), and other chemicals were acquired from Chengdu Kelong Chemical Co., Ltd. (Sichuan, China). Melamine–urea–formaldehyde (MUF) resin with a molar ratio of 0.04 : 1 : 1.14 (melamine/urea/formaldehyde) was synthesized by the laboratory. The 20 mm thick eucalyptus veneers were acquired from Xiangyi wood industry Co., Ltd. (Sichuan, China).

### 2.2. Preparation of MUFP

The MUFP with a molar ratio of 0.07 : 1 : 2.17 (melamine/urea/formaldehyde) was prepared as follows: 255 g formaldehyde aqueous solution (37 wt%) and a stirrer were charged into three-necked flask. The pH value of the reaction solution was maintained at 8.5 with 20% NaOH solution throughout the preparation process, and the temperature of reaction solution was gradually raised through a water bath. When the temperature reached 50 °C, 75 g urea and 13 g melamine were added into reaction solution, and the pH value was kept at 8.5. The solution temperature was then raised to 90 °C and kept for 30 minutes. Following this step, 12 g urea was charged into the reaction solution, and the reaction process was terminated after 20 minutes. Finally, the solution was freeze-dried to remove most of the water and formaldehyde, and MUFP solid was obtained.

### 2.3. Preparation of various adhesives

Table 1 lists the formulation of each adhesive. 10 g soy protein isolate and 90 g distilled water were charged into flask stirring with a mechanical stirrer to prepare soy protein adhesive (SPI adhesive). The slurry was stirred at 50 °C for 30 minutes and kept its pH value at 9.

**Table 1:** Various adhesive formulations

Adhesives	Formulations
SPI	Soy protein isolate (10 g); distilled water (90 g)
SPI/MUFP-1	SPI adhesive (100 g); 1% MUFP (1 g); distilled water (1 g)
SPI/MUFP-2	SPI adhesive (100 g); 2% MUFP (2 g); distilled water (2 g)
SPI/MUFP-3	SPI adhesive (100 g); 3% MUFP (3 g); distilled water (3 g)
SPI/MUFP-4	SPI adhesive (100 g); 4% MUFP (4 g); distilled water (4 g)

To prepare a series of modified adhesives (SPI/MUFP adhesive), different amounts of MUFP (Table 1) were added to SPI adhesive after being dissolved in distilled water of the same quality, and the slurry was stirred rapidly for 20 minutes at 20 °C.

### 2.4. Testing of shear strengths of plywood

To prepare a three-layer plywood, various adhesives were coated on the veneer with a coating rate of 330 g m^−2^ 30% flour was added into MUF resin before coating. After assembling, three veneers were hot-pressed at 130 °C and 1.2 MPa for a hot-pressing time of 70 s mm^−1^. The resulting plywood was tested after being placed for 24 hours.

**Fig. 1:**
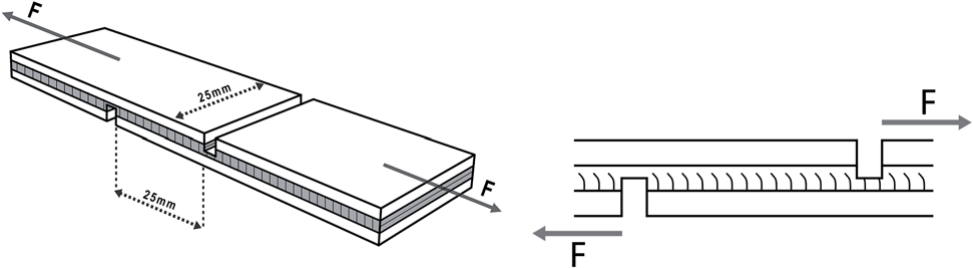
Specifications of plywood sample for the determination of shear strengths.

The specifications of the sample used for shear strength testing are shown in Fig. 1, and the tensile direction should be consistent with the plan view in the Fig. 1 to avoid lathe checks from affecting the shear strength.^35^ To determine the shear strengths of plywood, the samples were stretched to be damaged by the mechanical testing machine at a speed of 10 mm min^−1^, and the maximum tensile force was recorded. Moreover, samples for the determination of wet shear strength were soaked at 63 °C for 3 hours before the test. The shear strengths values were calculated by the following formula. The average value of shear strength was obtained *via* 24 tests.1
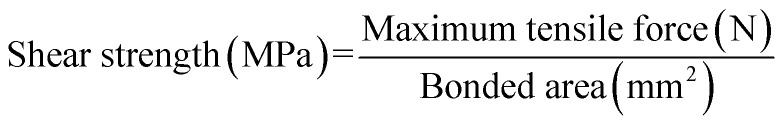


### 2.5. Testing of adhesive performance

The viscosity of the adhesives was determined by a NDJ-5S rotary viscometer (Shanghai Yoke Instrument Co., Ltd., Shanghai, China). All adhesive samples were tested three times at 20 °C by spindle No. 4 at 30 rpm.

To measure the residual rate of adhesive, the adhesives were completely cured in an oven at 130 ± 2 °C and milled to 100 mesh powder. The powder with a weight of *m* g was wrapped by a filter paper with a weight of *n* g. The filter paper containing the sample was soaked in 500 mL of distilled water at 60 °C for 6 hours, and then it was washed several times with 1 L of distilled water to filter out attachments. After sufficient washing, it was fully dried at 103 °C for 3 hours and its weight (*M* g) was recorded. The residual rate of adhesive was calculated by formula (2).2
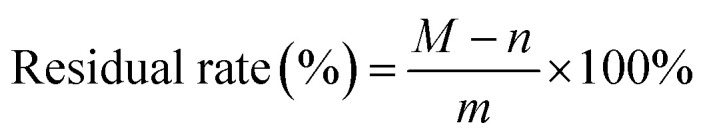


### 2.6. FT-IR spectroscopic analysis

Each fully cured adhesive was dried and milled to powder of less than 200 mesh. 1 g KBr and 0.01 g sample powder were well mixed to prepare a pellet. All specimens were analyzed by a Nicolet 670 spectrometer (Nicolet Instrument Co., USA). The spectrum of each sample with a resolution of 2 cm^−1^ was obtained by scanning 32 times, and the wavenumber range of 400 to 4000 cm^−1^ of each spectrum was selected.

### 2.7. GPC analysis

MUFP and MUF resin were freeze-dried to remove moisture and formaldehyde, and then they were dissolved in *N*,*N*-dimethylformamide (chromatographically pure) at a concentration of 2 mg mL^−1^. The molecular weight of each sample was measured by Waters GPC 1515 (Waters Co., USA) with the Agilent PLgel 5um MIXED-C chromatographic column (*M*_w_ range: 500 to 2 000 000). Each sample solution that had been placed for 24 hours was shaken until it was uniform, and then it was injected into the column. The flow rate was 1 mL min^−1^ and the mobile phase was *N*,*N*-dimethylformamide (chromatographically pure). The molecular weights of the standard samples (polystyrene) were 580, 1390, 4830, 9970, 29 150, 69650,152 600, and 224 900, respectively.

### 2.8. TGA

Each fully cured adhesive was dried and milled to powder of less than 200 mesh. The thermal stabilities of all samples were measured by the TG209F1 (NETZSCH Co., Selb, Germany) in a nitrogen environment. About 5 mg of each sample was put into a crucible and heated at a rate of 10 °C min^−1^. The weight loss values of each adhesive in the range of 50 to 600 °C were recorded.

### 2.9. SEM analysis

Each fully cured adhesive was dried and ground. The samples coated with gold film were imaged by a JSM-7500F field emission scanning electron microscope (JEOL Ltd., Tokyo, Japan) at 5 kV.

## 3. Results and discussion

### 3.1. FT-IR spectroscopic and GPC analyses

**Fig. 2:**
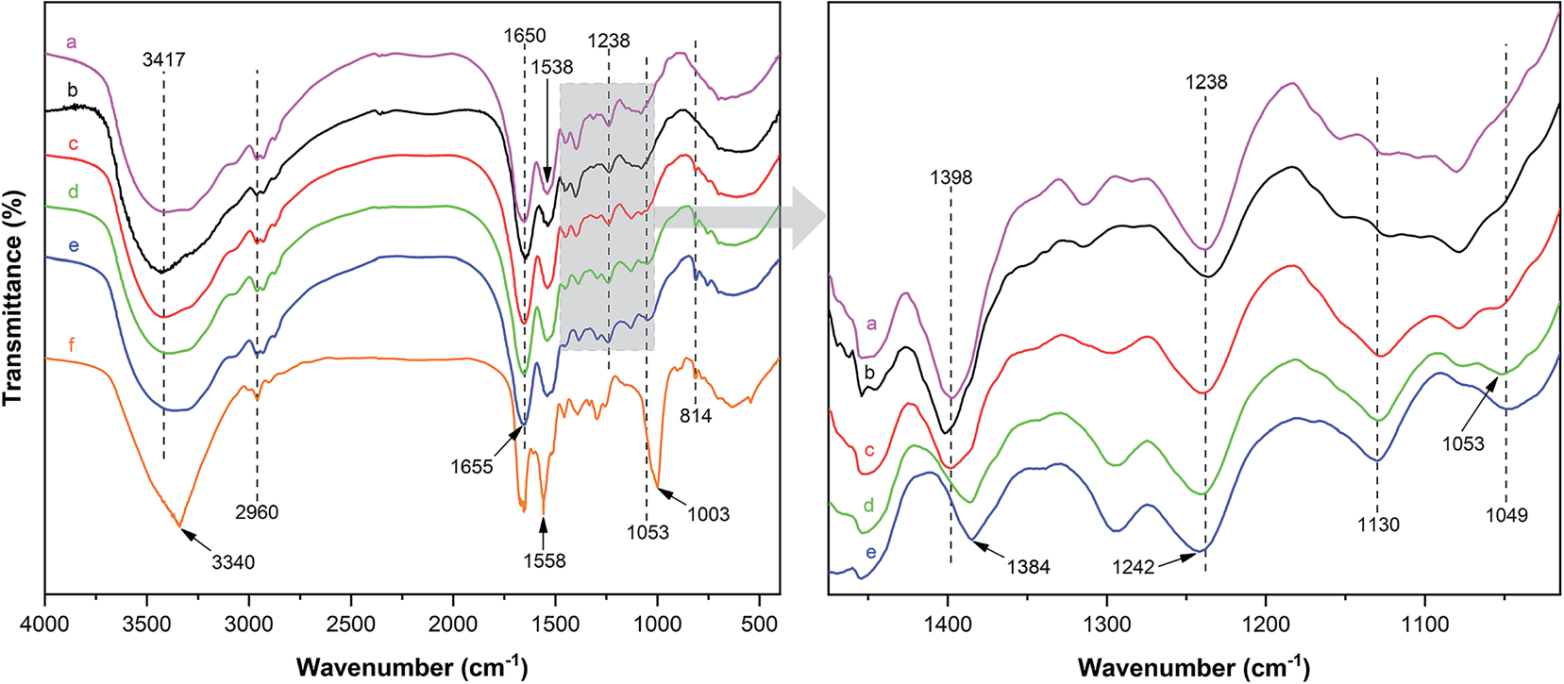
FT-IR spectrum of MUFP, SPI adhesive, and SPI/MUFP adhesives: (a) SPI adhesive; (b) SPI/MUFP-1 adhesive; (c) SPI/MUFP-2 adhesive; (d) SPI/MUFP-3 adhesive; (e) SPI/MUFP-4 adhesive; (f) MUFP.

**Fig. 3:**
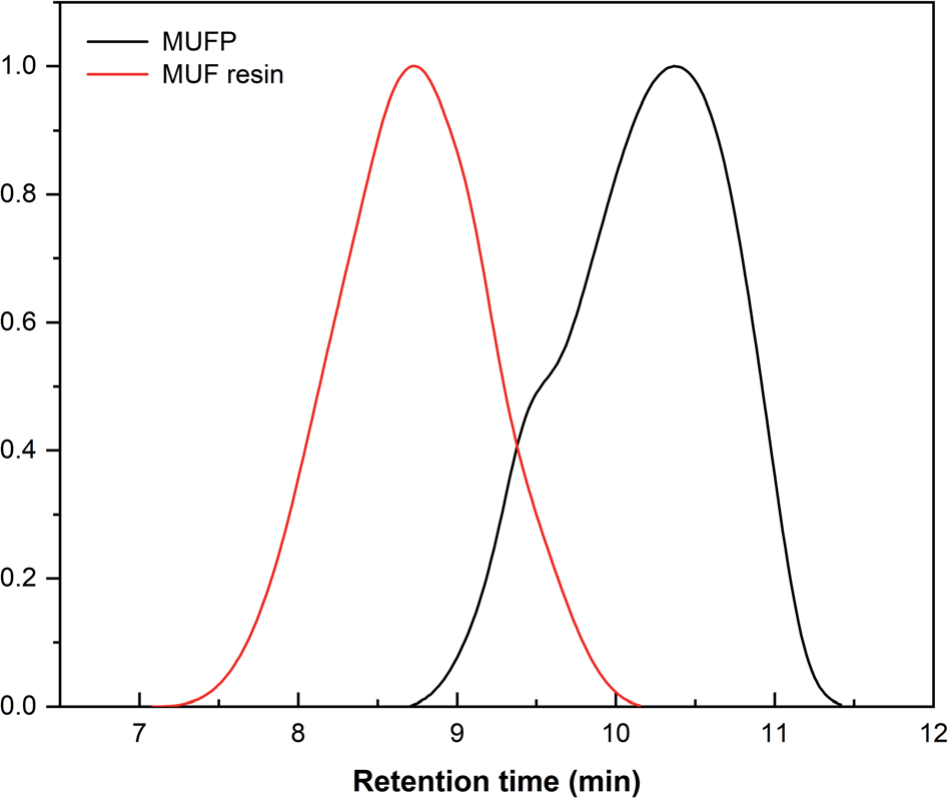
GPC spectrum of MUFP and MUF resin.

**Fig. 4:**
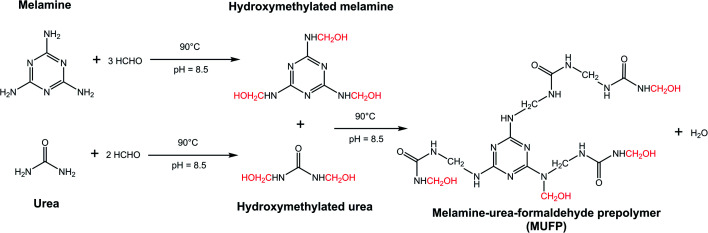
Synthesis process of melamine–urea–formaldehyde prepolymer (MUFP).

Fig. 2 illustrates the FT-IR spectrum of MUFP, SPI adhesive, and SPI/MUFP adhesives. In the FT-IR spectrum of MUFP (Fig. 2f), the peak at 3340 cm^−1^ was attributed to the presence of N–H and O–H groups.^36^ The peaks at 1655 and 814 cm^−1^ were assigned to C=O stretching and the triazine ring out-of-plane vibrations, respectively.^29^ Further, the weak peak at 2960 cm^−1^ and the strong peak at 1003 cm^−1^ were assigned to stretching vibrations of –CH_2_– and bending vibrations of the C–O from hydroxymethyl groups (–CH_2_OH), respectively.^29,32^ The results indicated that urea and melamine were successfully hydroxymethylated. Moreover, according to the GPC spectrum (Fig. 3) and Table 2, the *M*_n_ and *M*_w_ values of MUFP were 1066 and 2325, respectively, indicating that polycondensation reactions had occurred between hydroxymethylated urea and hydroxymethylated melamine, and the N–CH_2_–N groups were formed. In addition, the *M*_n_ and *M*_w_ values of MUF resin were about 15 times of those of MUFP, and the distribution index of MUFP was greater than 2. These results further indicated that the degree of polymerization of MUFP was much lower than that of MUF resin, and MUFP is mostly small molecule. The synthesis process of MUFP is shown in Fig. 4.

**Table 2:** *M*_n_, *M*_w_, and distribution index of MUFP and resin

Samples	M_*n*_	M_*w*_	Distribution index (*D* = *M*_w_/*M*_n_)
MUFP	1066	2325	2.181
MUF resin	15 599	35 473	2.274

In the FT-IR spectrum of SPI (Fig. 2a), the peaks at 1650, 1538, and 1238 cm^−1^ correspond to amide I (C=O stretching vibration), amide II (N–H bending vibration), and amide III (N–H and C–N stretching vibration), respectively.^37,38^ The absorption band at 3417 cm^−1^ was attributed to the free and bound N–H and O–H stretching vibrations of the amide and hydroxyl groups,^37^ and the peak at 1398 cm^−1^ was assigned to the C–O stretching vibration in the COO– group of protein molecules.^39–41^

**Fig. 5:**
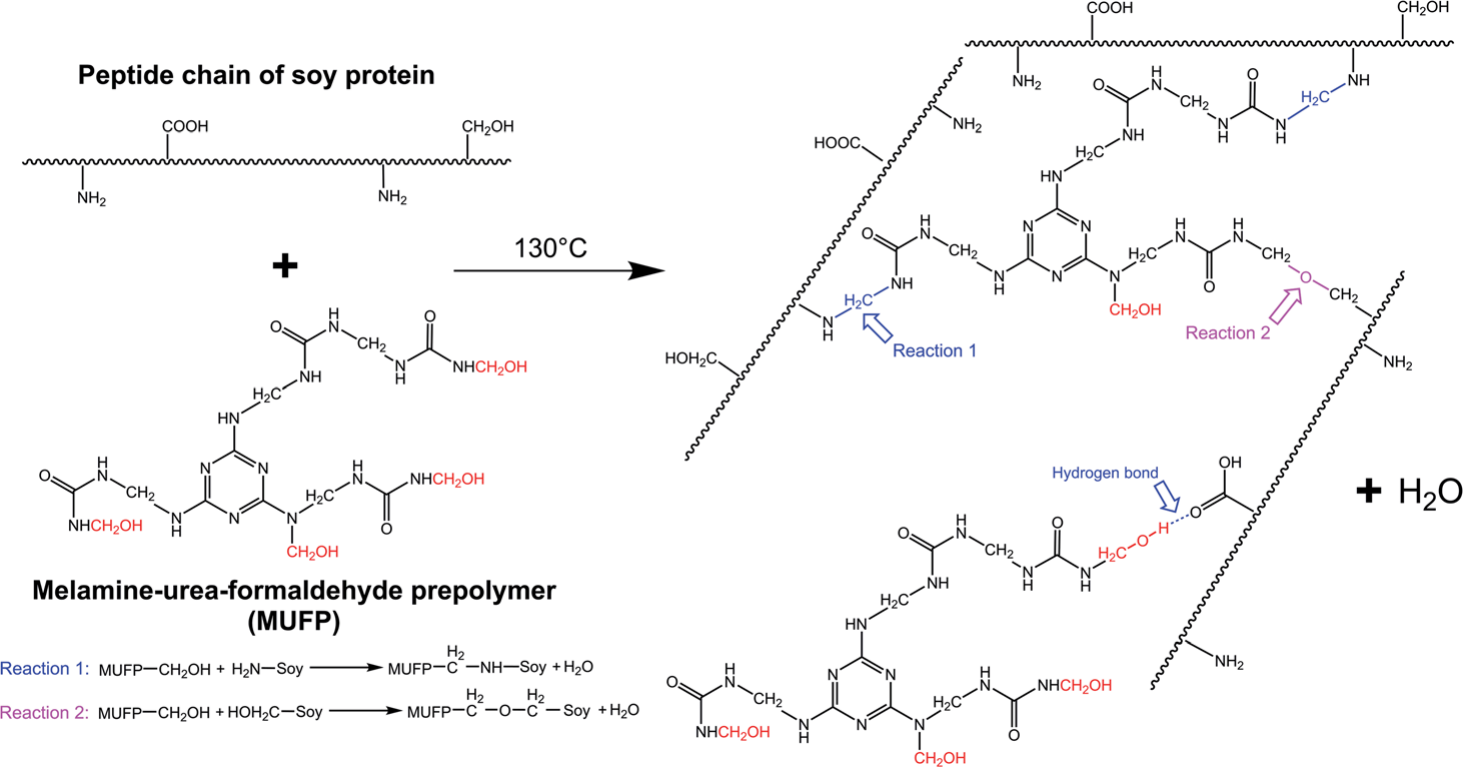
Cross-linking reaction process between the soy protein isolate and melamine–urea–formaldehyde prepolymer (MUFP).

With an increase in the amount of MUFP additive from 0 to 4%, the peak for the triazine ring vibrations (814 cm^−1^) gradually increased, suggesting that MUFP was uniformly dispersed in the adhesives. After adding MUFP, a new peak corresponding stretching vibrations of C–O–C was observed at 1130 cm^−1^,^29^ and the peaks of amide I and amide III shifted from 1650 to 1655 cm^−1^ and 1238 to 1242 cm^−1^ (blue shift) respectively. The bule shift of the amide peak was due to the fact that the structure of molecules became more stable and group vibrations required more energy.^42,43^ Thus, these changes indicated that the hydroxymethyl groups of MUFP reacted with the reactive groups (–NH_2_, –CH_2_OH) of peptide chains to increase the cross-linking density of the adhesive. In addition, when the amount of MUFP additive was increased to 3%, a new peak was observed at 1053 cm^−1^ (C–O of –CH_2_OH bending), suggesting there was an excessive amount of hydroxymethyl groups which did not participate in the reaction in SPI/MUFP-3 adhesive. Furthermore, as the amount of MUFP additive was increased from 2% to 4%, the peaks for the COO– and –CH_2_OH shifted 1398 to 1384 cm^−1^ and 1053 to 1049 cm^−1^ (red shift) respectively, indicating that the excessive hydroxymethyl groups of MUFP formed a large number of intermolecular hydrogen bonds with the reactive groups of protein molecule. The cross-linking reaction process is shown in Fig. 5.

### 3.2. TGA

**Fig. 6:**
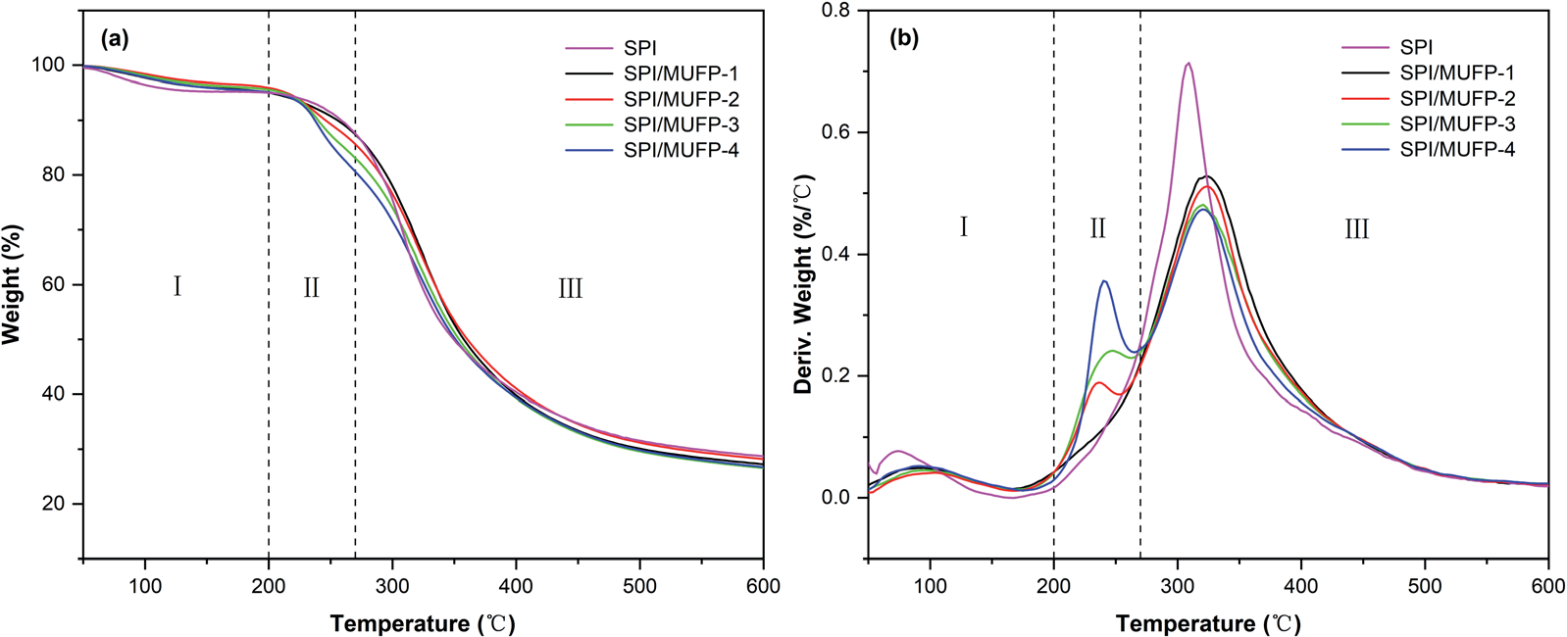
TG and DTG curves of different cured adhesives: (a) TG; (b) DTG.

Fig. 6 illustrates the thermogravimetric (TG) and derivative thermogravimetric (DTG) of each adhesive. There were three principal stages in the thermal degradation process of SPI adhesives: 50–200, 200–270, and 270–500 °C. In the first stage, the residual moisture in the adhesive evaporated, resulting in a slight weight loss of less than 10%. In the second stage, the breaking of unstable chemical bonds and the degradation of some micromolecular compounds caused major weight loss. In the third stage, degradation of the main protein skeleton structure led to weight loss. Table 3 summarizes the weight loss values.^44–46^

**Table 3:** Maximal degradation peak temperature and weight loss features of different adhesives

Adhesives	Maximal degradation peak temperature (°C)		Weight loss features (%)		
	Stage II	Stage III	Stage I	Stage II	Stage III
SPI	—	309.2	4.4	7.3	56.2
SPI/MUFP-1	—	322.3	4.8	7.6	57.3
SPI/MUFP-2	236.3	323.8	3.9	10.1	54.6
SPI/MUFP-3	248.3	320.8	4.3	12.2	53.8
SPI/MUFP-4	240.0	320.0	4.7	14.5	50.8

In the DTG curve, the curve of SPI adhesive featured only one degradation peak at a temperature of 309.2 °C in the third stage. When 1% MUFP was introduced into the adhesive, no new peak was observed and the peak temperature in the third stage increased to 322.3 °C, indicating that the chemical cross-linking reaction occurred between MUFP and peptide chains, and the cross-linking structure enhanced the thermal stability of the adhesive,^47^ which was consistent with the results of FT-IR analysis. Further, when the amount of MUFP additive was increased from 1% to 2%, the peak temperature in the third stage increased to 323.8 °C, suggesting that the density of the cross-linking structure was further enhanced. Additionally, as the amount of MUFP added was further increased from 2% to 4%, a new peak appeared and an increase in weight loss were observed in the second stage, and the peak temperature in the third stage was decreased to 320.0 °C. Combined with FT-IR analysis, the reasons for this finding are as follows: when the amount of MUFP additive reached 3%, the content of MUFP was excessive, and the excessive MUFP could not react chemically with protein molecules. Although the intermolecular hydrogen bonds between the excessive MUFP and protein molecules could further increase the cross-linking density of the adhesive structure, these excessive low-molecular-weight MUFP and hydrogen bonds were easily degraded in the second stage, leading to a decrease in the thermal stability of the adhesive.

### 3.3. Viscosity and residual rate analysis

The spreading and penetrating ability of the adhesive is mainly determined by its viscosity. Adhesives with very high viscosity cannot be spread evenly on the board, whereas adhesives with very low viscosity are difficult to stay on the surface of the board to form a glue layer.^22^Table 4 shows the viscosity of different soy protein adhesives. The viscosity value gradually increased from 5660 to 18 169 mPa s when the amount of MUFP additive was increased from 0 to 4%. This change may be due to the intermolecular hydrogen bonds formed by the hydroxymethyl groups of MUFP and the hydrophilic groups of peptide chains that caused an increase in the friction among molecules.^48,49^

**Table 4:** Viscosity of different adhesives and residual rate of different cured adhesives

Samples	Viscosity (mPa s)	Residual rate (%)
SPI	5660 ± 62	70.69 ± 0.55
SPI/MUFP-1	7767 ± 40	80.59 ± 0.97
SPI/MUFP-2	10 625 ± 67	84.38 ± 0.84
SPI/MUFP-3	14 346 ± 66	82.72 ± 0.54
SPI/MUFP-4	18 169 ± 108	79.57 ± 0.86

After the adhesive is soaked, water-soluble substances and oligomers that have an adverse impact on water resistance of the adhesive will be filtered out, so residual rate is an effective parameter to evaluate the water resistance of adhesive.^25,45^ The values of resistance rate reflecting the water resistance property of the adhesives are summarized in Table 4. The addition of MUFP significantly improved the residual rate (13.69%) of soy protein adhesives, indicating that the introduction of MUFP improved the water resistance of the adhesive. This was due to two reasons: (1) the number of hydrophilic groups in soy protein was reduced by reaction with the hydroxymethyl groups of MUFP, resulting in reduced solubility of the cured adhesive; (2) after the adhesive was cured, the covalent bonds produced by the cross-linking reaction replaced the water-intolerant intermolecular hydrogen bonds resulting in a rigid structure, thereby inhibiting water penetration. With the increase of additive amount, the residual rate was first increased and then decreased. The residual rate reached the maximum value, when the additive amount was 2%. That was because MUFP is hydrophilic, when the amount of MUFP added was more than 2%, the excessive MUFP led to a decrease in the residual rate, which conformed to the results of TGA.

### 3.4. SEM analysis

**Fig. 7:**
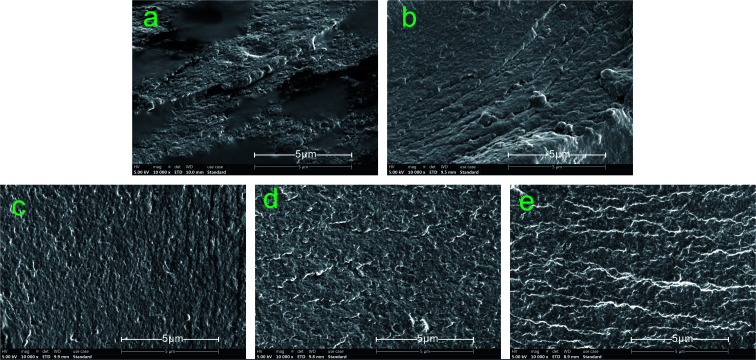
Fracture surface micrographs of different cured adhesives: (a) SPI adhesive; (b) SPI/MUFP-1 adhesive; (c) SPI/MUFP-2 adhesive; (d) SPI/MUFP-3 adhesive; (e) SPI/MUFP-4 adhesive.

Fig. 7 shows the fracture surface micrographs of the different cured adhesives. Many cracks and holes were detected on the fracture surface of SPI due to the evaporation of moisture during the curing process, which increased the permeability of water in the adhesive and caused a decrease in the water resistance of the adhesive.^50^ Moreover, the fracture surface of SPI was very rough, revealing the low internal bonding force of the SPI adhesive.^51^ The number of cracks and holes on the surface were reduced and the fracture surface became smoother when the MUFP was introduced into adhesives (Fig. 7b and e), and the surface was smoothest and had the least number of cracks when the additive amount was 2%.

When the amount of MUFP additive was increased from 0 to 2%, the number of cracks and holes was gradually reduced and the fracture surface became smoother. This finding suggested that the chemical cross-linking reactions occurred between MUFP and peptide chains, which improved the cohesive force of the adhesive and make the structure of cured adhesive more orderly and compact, resulting in an increase in the water resistance. However, as the amount of MUFP additive was further increased from 2% to 4%, the adhesive surface remained smooth but the number of cracks was increased, indicating that the water resistance of the adhesive was decreased. These changes may be due to excessive MUFP filling the voids of the adhesive structure, affecting the water evaporation, and increasing the internal stress of the adhesive.^33^

### 3.5. Shear strength analysis

**Fig. 8:**
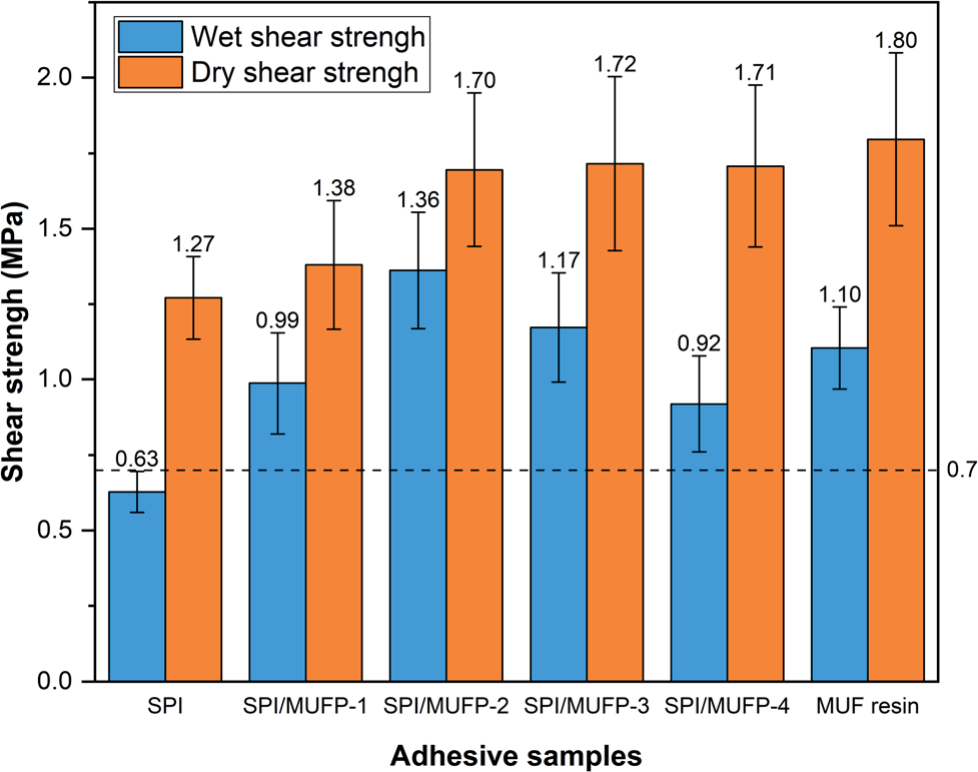
Dry and wet shear strengths of plywood prepared by different adhesives.

Fig. 8 illustrates the dry and wet shear strengths of plywood prepared by various adhesives. The wet shear strength is determined by the bonding strength and water resistance of the adhesive.^52^ Evidently, the wet shear strength of plywood prepared by the SPI adhesive did not reach the standard for plywood type II (the Chinese National Standard GB/T 9846–2015, ≥0.7 MPa).

The wet shear strength of the plywood prepared by all SPI/MUFP adhesive met the requirement of plywood type II, and the maximum value was 1.36 MPa when the amount of MUFP additive was 2%. Moreover, the wet shear strength of SPI/MUFP-2 adhesive was 23.6% higher than that of MUF resin. Based on TGA and SEM analysis, there were two causes for explaining this remarkable improvement. (1) The bonding force of the SPI adhesive was mainly provided by the physical entanglement and weak intermolecular interaction among protein molecules formed during the curing process, while the covalent bonds produced by the cross-linking reactions between MUFP and peptide chains provided a stronger bonding force for SPI/MUFP adhesives.^41^ (2) The cross-linking reactions between MUFP and peptide chains formed a denser network structure in the adhesive, blocking more water from entering the adhesive. However, as the amount of MUFP additive was increased from 2% to 4%, the wet shear strength was decreased by 32.4% but the dry shear strength was hardly changed, reflecting the dramatically decrease in the water resistance of the adhesive, conforming to the results of residual rate determination. According to the FT-IR and SEM analyses, this decrease was attributed to two reasons. Firstly, the presence of excessive MUFP with massive hydrophilic groups (hydroxymethyl) would attract more water into the adhesive. Secondly, one of the main sources of bonding force was the massive intermolecular hydrogen bonds formed between the hydroxymethyl groups on excessive MUFP and the hydrophilic groups of proteins, which were easily destroyed in humid environment, resulting in low water resistance of adhesives.

The dry shear strength of the plywood boned by SPI/MUFP-3 adhesive was the maximum (1.72 MPa) among all soy protein adhesives, and the dry shear strength of plywood prepared by SPI/MUFP-4 (1.71 MPa) was higher than that of plywood prepared by SPI/MUFP-1, contrary to the result of wet shear strength. This was because the hydrogen bonds formed by excessive MUFP could further provide effective bonding force in a dry environment. Nevertheless, as the amount of MUFP added was increased from 2 to 4%, the improvement in dry bonding was not obvious. This may be due to the excessive increase in the viscosity of the adhesive which is not conducive to the penetration of the adhesive into the wood to form a mechanical interlocking structure.

## 4. Conclusions

In this study, the MUFP with high reactivity was successfully synthesized, and different amounts of MUFP were used to ameliorate the performance of the soy protein isolate (SPI) adhesive. The results indicated that the method was environmentally-friendly and low-cost. When the amount of MUFP additive was 2%, MUFP significantly enhanced the water resistance (13.69%) and bonding quality of SPI adhesive. The wet shear strength of the plywood prepared by the modified adhesive were increased by 115.9%, respectively, compared to SPI adhesive, reaching the maximum (1.36 MPa). Furthermore, the wet shear strength of SPI/MUFP-2 was 23.6% higher than that of MUF resin. FT-IR, ATGA, and SEM tests proved that the increase in bonding strengths was due to the chemical cross-linking reactions between the hydroxymethyl groups of MUFP and the peptide chains. However, when the amount of MUFP additive was higher than 2%, presence of excessive hydrophilic groups and hydrogen bonds would lead to a decrease in water resistance and thermal stability of adhesives.

## Author contributions

Conceptualization, H. X.; methodology, K. J., Z. L., and H. X.; software, K. J., M. Y., W. L., M. J., Q. F., H. T., and H. X.; validation, K. J., and Z. L.; formal analysis, H. X.; investigation, K. J., M. Y., W. L., M. J., Q. F., and H. T.; resources, K. J., M. Y., W. L., M. J., Q. F., H. T., and Y. C.; data curation, K. J. and Z. L.; writing—original draft preparation, H. X.; writing—review and editing, K. J. and Z. L.; visualization, H. X.; supervision, Z. L.; project administration, K. J.; funding acquisition, Y. C. All authors have read and agreed to the final version of the manuscript submitted for publication.

## Conflicts of interest

There are no conflicts to declare.

## Supplementary Material
